# Supplementary material for: Interface-driven formation of a two-dimensional dodecagonal fullerene quasicrystal
Source: Nat Commun. 2017 May 22;8:15367. doi: 10.1038/ncomms15367 (PMC5458153; doi:10.1038/ncomms15367)
Supplement: Supplementary Information — Supplementary Figures and Supplementary Table [file ncomms15367-s1.pdf]

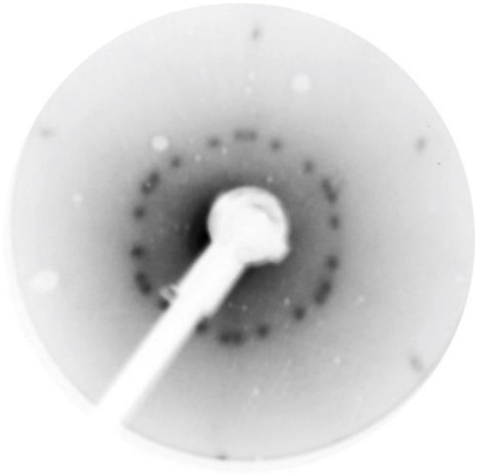

Supplementary Figure 1 | LEED pattern of fullerenes on 2Pt-Pt_3Ti(111). LEED pattern given in Figure 1 (c) showing the spots (without circles) of two hexagonal domains and the quasicrystalline domain.

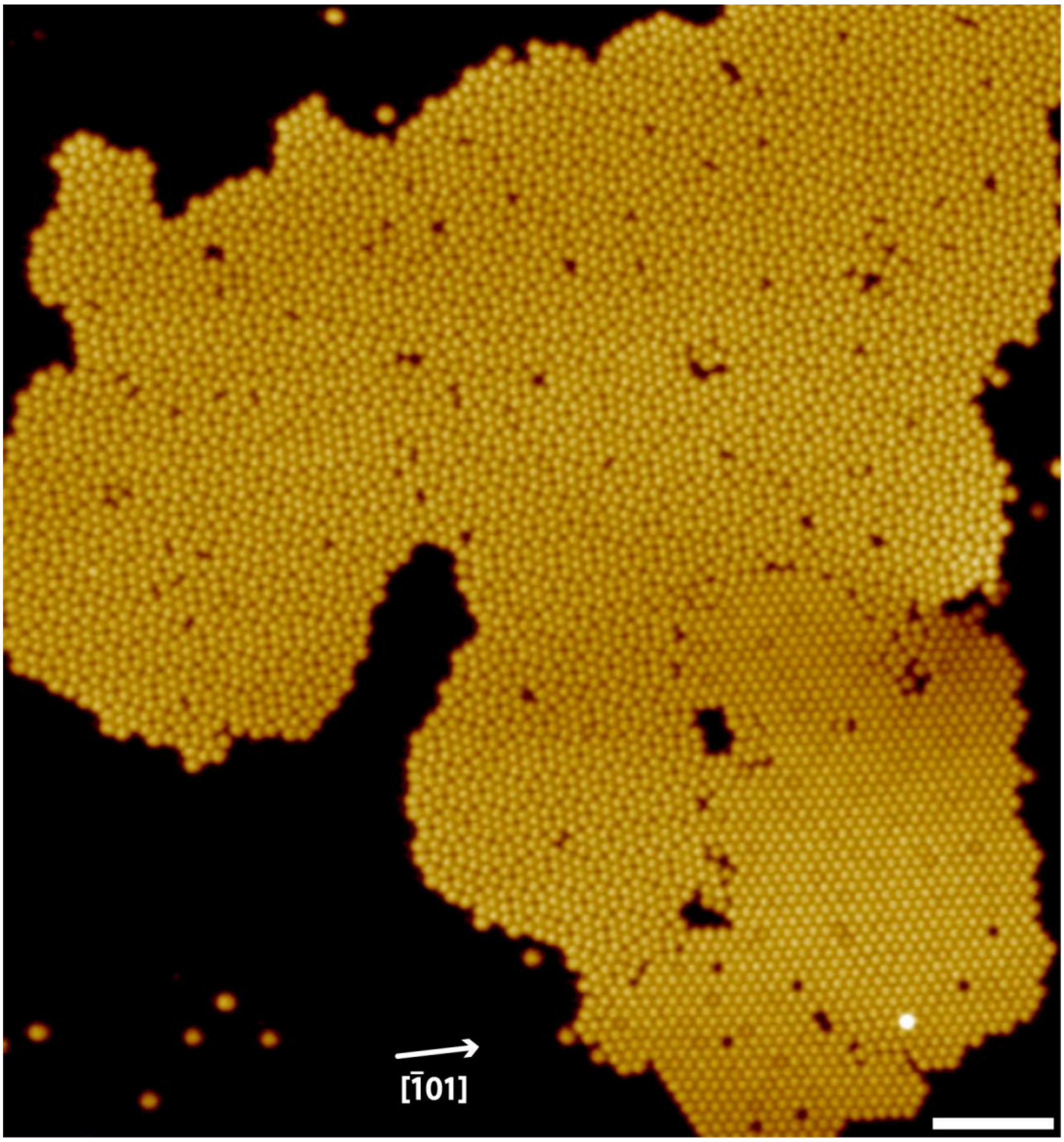

Supplementary Figure 2 | STM image of fullerenes on 2Pt-Pt_3Ti(111). Large area (scale bar: 10 nm) low temperature UHV-STM image showing a large 2D-quasicrystalline and a small hexagonal domaine of fullerenes (U_{set} = -2.42 V, I_{set} = 0.13 nA, 77 K).

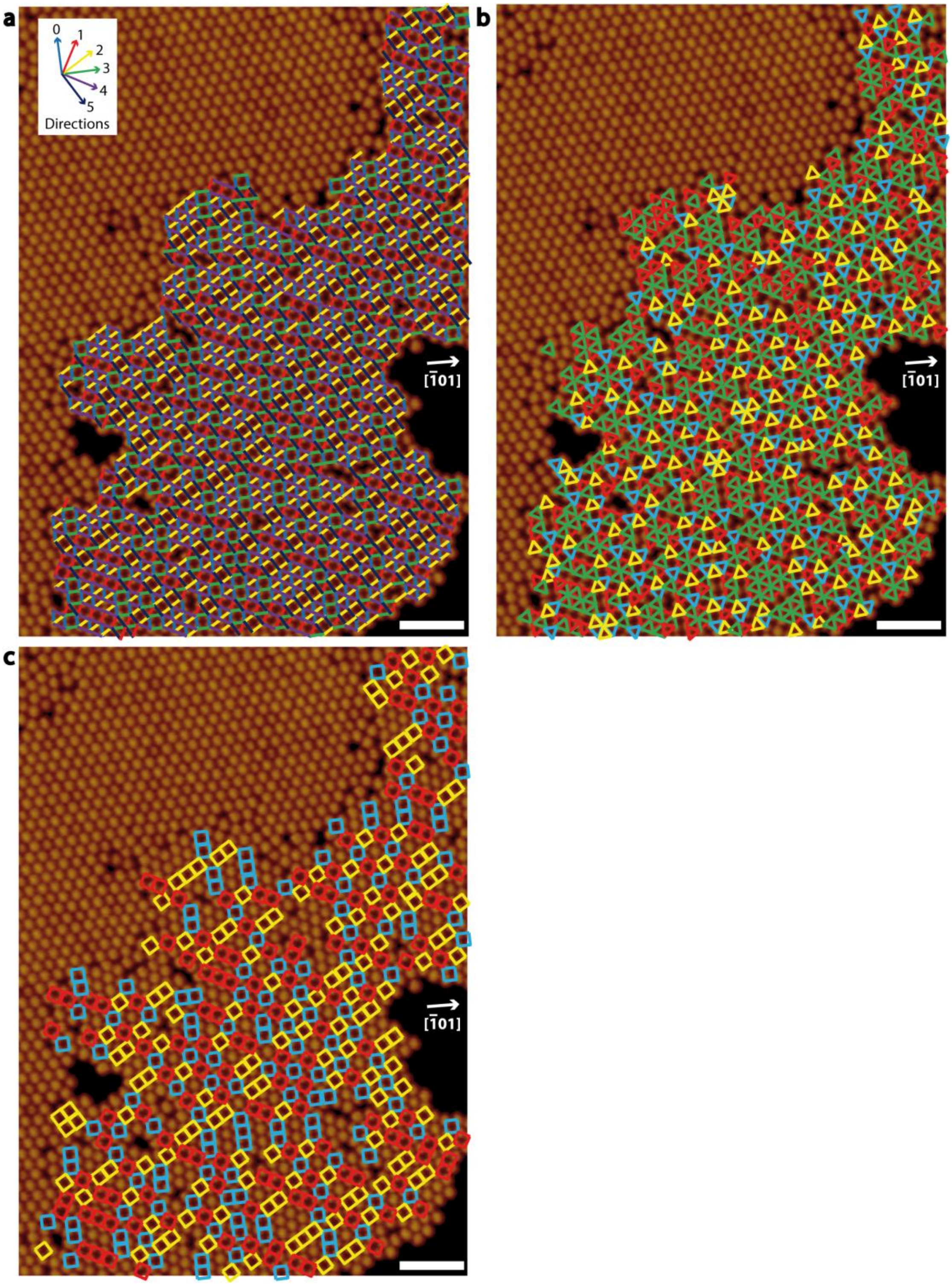

Supplementary Figure 3 | Statistical analysis of the self-assembled fullerene quasicrystal. **(a)** The dodecagonal tiling is superimposed onto the 2D-quasicrystalline structure of fullerenes on 2Pt-Pt_3Ti obtained from STM imaging (scale bar: 5nm, U_{set} = -2.03 V, I_{set} = 0.47 nA, 77 K). The six different edges are displayed in different colors in the upper left corner. (**b), (c)** Triangles and squares are superimposed onto the fullerene quasicrystal.

Supplementary Table 1 | Statistical analysis of the 2D fullerene quasicrystal. Analysis of the quasicrystalline domain shown in Supplementary Figure 3. The proportion of triangles (red and green) with edges in the preferred <1 -2 1> directions (directions 0, 2, and 4 in Supplementary Figure 3) corresponds to about 72 % of all triangles, while others (blue and yellow triangles) represent only 28 %. The squares are uniformly distributed among the three possible directions within the 12-fold symmetry, since they are composed of edges in both directions (<1 -2 1> as well as <-1 0 1>). Based on this statistical analysis of the self-assembled quasicrystalline domain of fullerenes on 2Pt-Pt_3Ti(111), the triangle to square ratio $R_{tr/sq}$ is 2.67.

**Direction**	**0**	**1**	**2**	**3**	**4**	**5**
**Edges**	582	324	585	313	590	307
**Squares**	147 (blue)	146 (red)	147 (yellow)			
**Triangles**	166 (blue)	422 (red)	160 (yellow)	426 (green)		

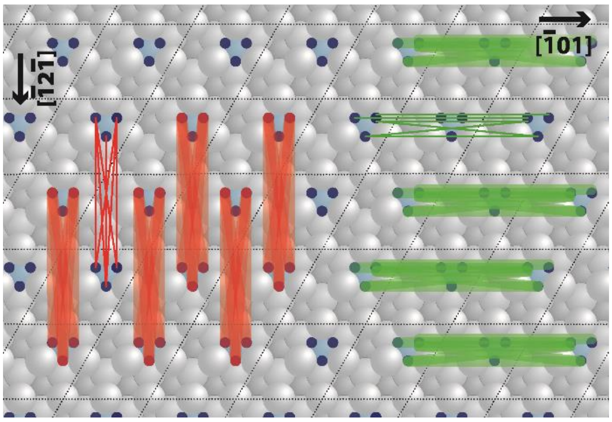

Supplementary Figure 4 | Schematic showing adsorption positions on 2Pt-Pt_3Ti(111). Based on the calculated adsorption energies, the threefold degenerate, favored bridge1 adsorption positions are indicated. The other adsorption sites are omitted for clarity. Narrow lines connect possible combinations of nearest neighbor positions in the [-1 2 -1] and [-1 0 1] directions within the distance range 0.956 nm to 1.1 nm, while expanded bars represent the corresponding bundle of lines in the respective direction. Six possible combinations of nearest neighbor positions are possible along <-1 0 1> directions, while seven combinations can be found along <1 -2 1> directions. The probability of finding bridge1 adsorption positions for pairs of fullerenes with an intermolecular line connection along <1 -2 1> directions is approximately twice that of finding such a pair along <-1 0 1> directions, as a result of the hexagonal symmetry of the substrate. This directionality of nearest neighbor positions is assumed to provide the reason for the smaller number of edges of triangles of the 2D QC in the <-1 0 1> directions.

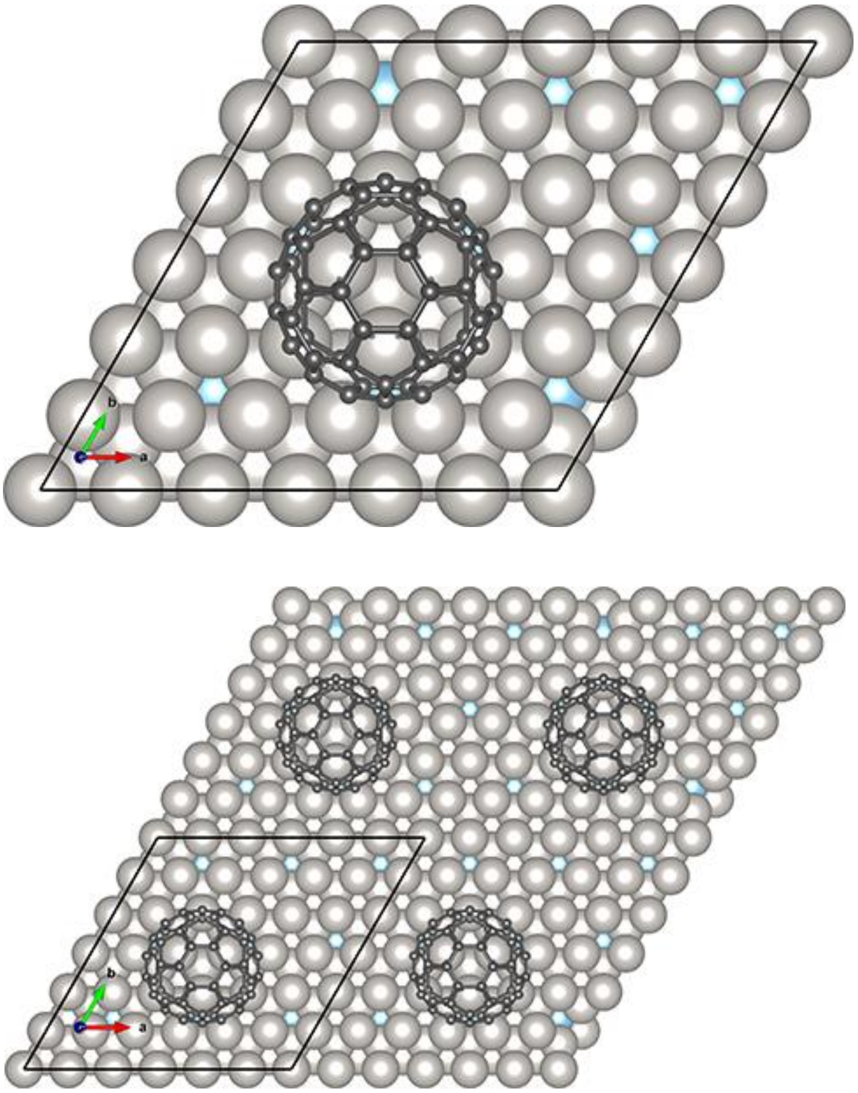

a

b

Supplementary Figure 5 | Hexagonal in-plane surface supercell investigated by DFT simulations. (**a**) Single C_{60} adsorbed in hcp position on 2Pt-$Pt_3Ti(111)$ with the hexagon parallel to the surface. The system was modeled by means of density functional theory (DFT) using a slab consisting of 5 atomic layers with a (3 x 3) hexagonal in-plane surface supercell. (**b**) Here four supercells are shown, illustrating the fullerene-fullerene separation of 1.675 nm.

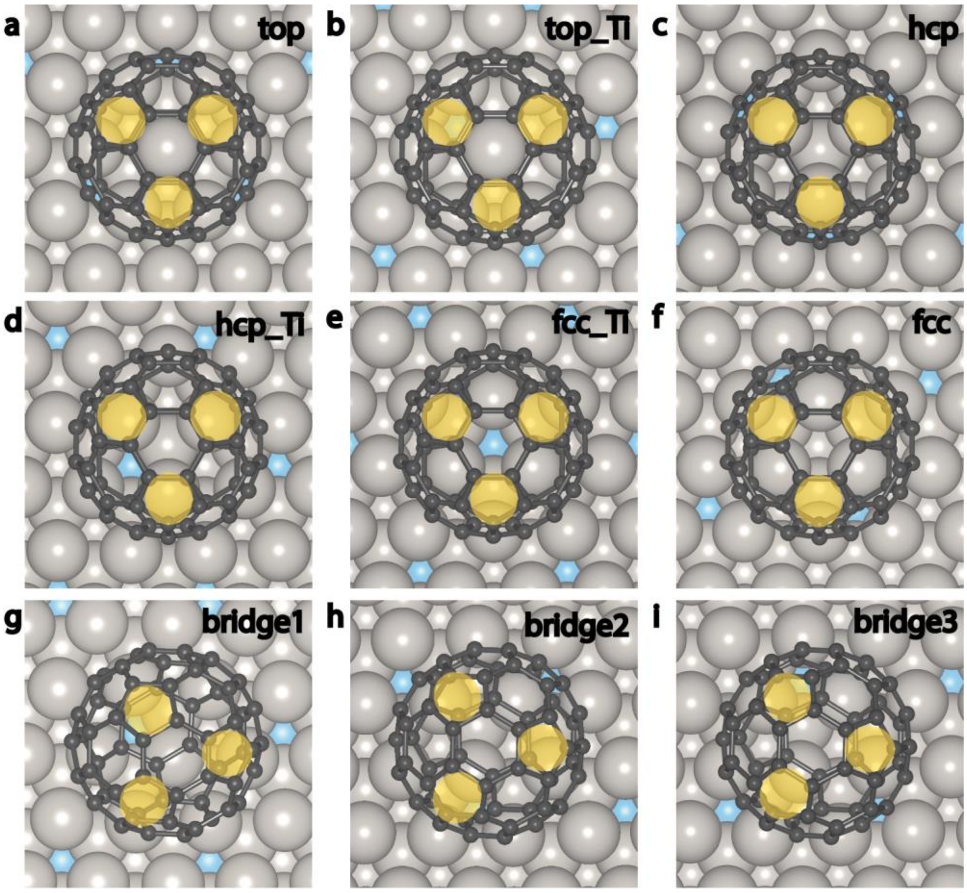

Supplementary Figure 6 | Adsorption configurations of C_{60} on 2Pt-Pt_3Ti(111). Schematic top view of adsorption configurations obtained using DFT calculations for nine different surface positions. While the lobes (yellow) of the three-lobe orbital structure of fullerenes adsorbed on bridge positions point along <-1 0 1> directions of the underlying substrate, the lobes of relaxed fullerenes on all other positions point along <1 -2 1> directions.

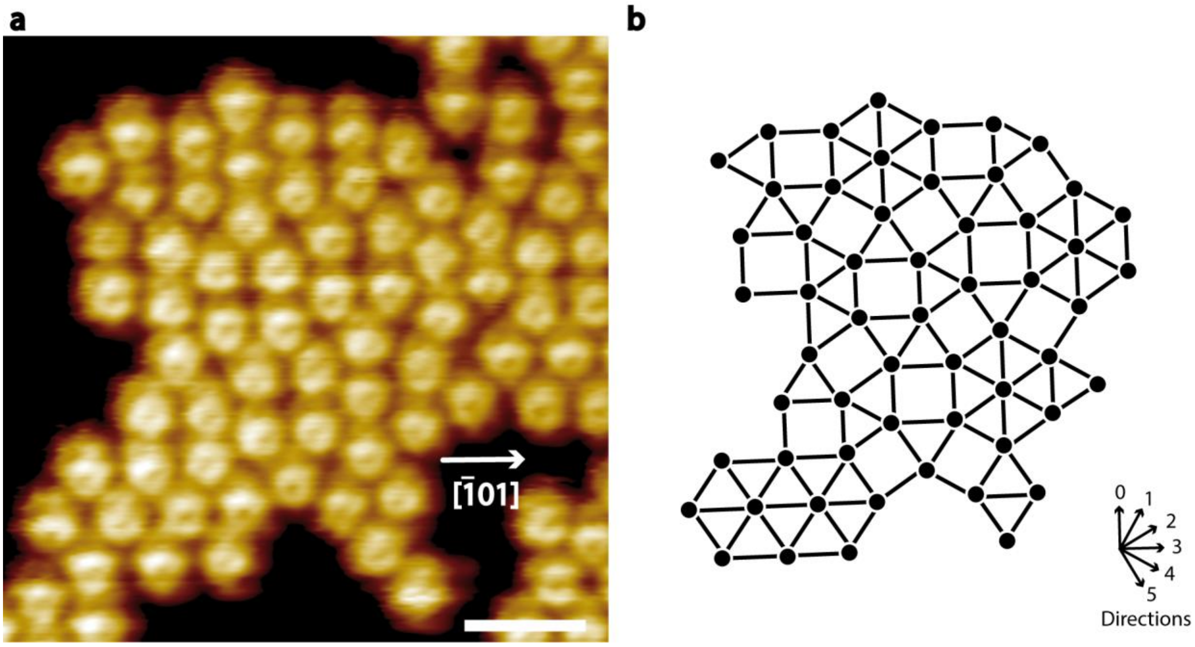

Supplementary Figure 7 | Dodecagonal tiling of fullerenes on Pt-Pt_3Ti(111). (a) Low temperature UHV-STM image showing a 2D-quasicrystalline structure of fullerenes (scale bar: 2 nm, U_{set} = -1.89 V, I_{set} = 0.16 nA, 77 K). (**b)** Triangle-square tiling extracted from (a).
